# Impacts of sheep versus cattle livestock systems on birds of Mediterranean grasslands

**DOI:** 10.1038/s41598-021-89975-x

**Published:** 2021-05-24

**Authors:** Rita F. Ramos, João A. Diogo, Joana Santana, João P. Silva, Luís Reino, Stefan Schindler, Pedro Beja, Angela Lomba, Francisco Moreira

**Affiliations:** 1 CIBIO (Centro de Investigação em Biodiversidade e Recursos Genéticos)/InBIO, (Rede de Investigação em Biodiversidade e Biologia Evolutiva), Universidade do Porto, Campus Agrário de Vairão, 4485-661 Vairão, Portugal; 2 CIBIO (Centro de Investigação em Biodiversidade e Recursos Genéticos)/InBIO, (Rede de Investigação em Biodiversidade e Biologia Evolutiva), Laboratório Associado, Instituto Superior de Agronomia, Universidade de Lisboa, Tapada da Ajuda, 1349-017 Lisbon, Portugal; 3 EAA, Environment Agency Austria, Spittelauer Lände 5, 1090 Vienna, Austria; 4 Community Ecology and Conservation Research Group, Faculty of Environmental Sciences, Czech University of Life Sciences, Prague, Czech Republic; 5 Cátedra EDP Biodiversidade/EDP Chair in Biodiversity, CIBIO/InBIO-Centro de Investigação em Biodiversidade e Recursos Genéticos, Universidade do Porto, Vairão, Portugal; 6 Cátedra REN em Biodiversidade, CIBIO/InBIO, Centro de Investigação em Biodiversidade e Recursos Genéticos, Laboratório Associado, Universidade do Porto, Campus Agrário de Vairão, 4485-661 Vairão, Portugal

**Keywords:** Agroecology, Grassland ecology, Environmental impact

## Abstract

Mediterranean pastures are experiencing strong changes in management, involving shifts from sheep to cattle-based livestock systems. The impacts of such shifts on biodiversity are still poorly understood. Here, we sought to contrast the grazing regime, vegetation structure, bird species richness and abundance, between sheep and cattle grazed parcels, to understand the mechanisms through which management decisions impact farmland birds. During spring 2019, we characterized livestock management, bird populations and sward structure in 23 cattle and 27 sheep grazed parcels. We used a Structural Equation Model to infer the direct and indirect effects of sheep and cattle grazing on birds. Although no effects were found on overall species richness, there were species-specific responses to sheep and cattle grazed systems. Grazing pressure (variable integrating stocking rate and the number of days in the parcel) had negative impacts on the prevalence/abundance of Zitting Cisticola, Corn Bunting and Little Bustard, either directly or indirectly, through the effects of grazing pressure on vegetation height. Animal density and vegetation cover had direct positive effects in *Galerida* spp. and Common Quail, respectively. Zitting Cisticola and Little Bustard also showed a direct response to livestock type. Our study emphasizes the importance of grazing pressure as a driver of negative impacts for bird populations in Mediterranean grasslands. Since the ongoing transition from sheep to cattle-based systems involves increases in stocking rate, and therefore potentially higher grazing pressure, we propose a policy change to cap the maximum allowed grazing pressure. At the landscape scale, a mix of sheep and cattle grazed fields would be beneficial for maintaining bird diversity.

## Introduction

The way grasslands are managed impacts their biodiversity and the potential for provisioning ecosystem service^1,2^. Understanding the mechanisms linking grassland management actions to biodiversity outcomes is therefore key to understand the impacts of existing or planned policies, and associated farmers’ decisions^3^.

Mediterranean grasslands are a stronghold for several farmland bird species of conservation concern^4–6^. The management of these farming systems has been changing in the last decades due to incentives from the European Union (EU) Common Agricultural Policy (CAP), which have been promoting the replacement of traditional dry cereal-based systems, including crop rotations and fallow land, by livestock-based systems associated to an increasing amount of permanent pastures^6–8^. The suitability of these pastures to farmland birds depends on management decisions including the livestock type, grazing pressure, timing of hay harvesting (when existing), or the application of fertilizers^9,10^. These will in turn impact on key drivers of bird populations, including vegetation structure, food resources, or disturbance levels^1,9,11^.

In the Iberian Peninsula, because of the persistence of CAP subsidies coupled to cattle (but less to sheep) production, and a highly subsidized beef-cattle production^10^, there has been a shift from sheep-based to cattle-based systems in these permanent pastures^7,10,12^. Previous studies have suggested that this management change is likely to differentially affect grassland birds through changes in sward structure and therefore habitat suitability for birds^10,13^, as well as increases in bird nest predation and trampling risk^14^.

Our overall aim was to contrast the implications and mechanisms of the cattle versus sheep management impacts on farmland bird populations in permanent pastures of the Iberian Peninsula. We focused on a High Nature Value region in Southern Portugal where the transition from traditional crop-based systems, which included sheep grazing in stubble fields and fallow land, towards livestock-based, mostly cattle, systems in permanent pastures has been occurring^7,10^. Previous studies in the area addressed the impact of field and landscape variables on bird populations, including grazing regimes, but were mostly focused on fallow parcels managed under a traditional farming system^13^ and did not address the mechanisms through which grazing impacted on birds. We sought to establish a connection between livestock management and bird populations through the characterization of the occurrence and density of breeding birds, vegetation structure and grazing pressure in a series of fields managed either for sheep or cattle. Subsequently, using a modelling approach, we explored the potential direct and indirect effects of livestock on birds’ density and occurrence. Direct effects of livestock management on birds were expected to occur either by differences in livestock type, resulting in behavioural differences with impacts on disturbance, trampling or nest predation^9,14^. Indirect effects were expected through impacts on vegetation, leading to changes in habitat quality and food resources that ultimately influence birds’ occurrence and density. Our main questions were: (a) how grazing regimes (animal density and duration of grazing) and vegetation structure differed between sheep versus cattle systems?; (b) how bird species richness, occurrence and density varied across livestock systems?; and, (c) what were the direct and indirect effects of livestock type on bird populations?

## Methods

### Study area and parcel selection

**Figure 1:**
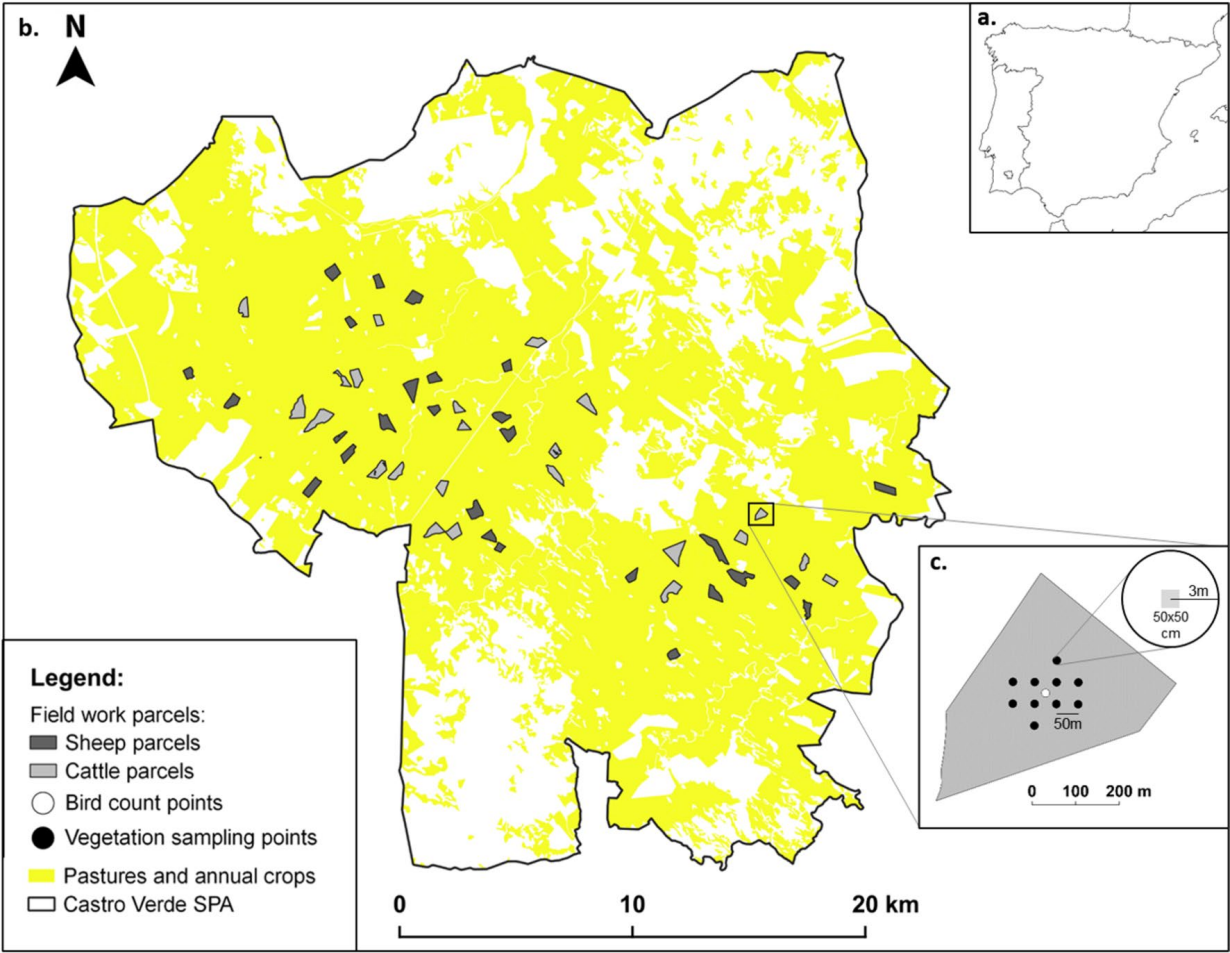
(**a**) Location of the study area within the Castro Verde Special Protected Area (SPA), southern Portugal. (**b**) Distribution of the 27 sheep (dark grey polygons) and 23 cattle (light grey polygons) grazing parcels and (**c**) Sampling scheme applied to each parcel surveyed. Bird counts were done at the centroid of the parcel (white dot) whereas vegetation sampling was performed at the indicated 10 points (black dots). The area covered with pastures and annual crops (derived from CORINE land cover 2018—https://land.copernicus.eu/pan-european/corine-land-cover/clc2018) is shown in yellow. The map was done using the version 3.10.0 of QGIS—https://qgis.org/en/site/index.html.

The study was conducted in Castro Verde Special Protection Area (SPA), located in southern Portugal (Fig. 1). The climate is Mediterranean, with hot summers (30–35 °C on average in July) and mild winters (averaging 5–8 °C in January), and over 75% of annual rainfall (500–600 mm) concentrated in October–March. The landscape is flat or gently undulating (100–300 m), mainly dominated by open areas used for rainfed pastures (ca. 60%) and annual crops (ca. 25%), and to a less extent by open woodlands (ca. 7%)^15^.

Since 1995, part of the study area has benefited from a CAP agri-environment aiming to protect the traditional farming system^16^. This scheme provides financial support to farmers for agricultural practices considered favourable to conservation, including the traditional rotation of cereals and fallows, the maintenance of low stocking rates (usually related with sheep grazing systems), and sowing of crops benefiting grassland birds^16^. However, in recent years the traditional farming system has been declining, with many farmers converting to specialized livestock systems, mainly, cattle grazing systems, with an increase of stocking rates^7,15^.

Parcel selection started by identifying grasslands grazed by either sheep or cattle, based on parcel-level statistical information from 2010 provided by the Portuguese Ministry of Agriculture^7^. To minimize potentially confounding effects of adjacent land uses (edge effects) and other non-crop elements within parcels on bird assemblages, we excluded parcels less than 100 m from shrubland or forested areas, with shrub and tree cover > 5% and with a minimum size of 10 ha. In January 2019 we visited 100 pre-selected parcels which were grazed by either sheep or cattle in 2010 in order to confirm the parcel land use in the agricultural year of 2018/2019, aiming to sample a balanced proportion of 50 sheep and cattle grazed parcels. Additional livestock information for the agricultural year of 2018/2019 was obtained during systematic visits to targeted parcels (see “Grazing Regime” section from Methods). We ended up with 23 cattle parcels and 27 sheep parcels (Fig. 1).

### Bird and vegetation data

Breeding birds were sampled twice in each parcel during 7–16 April and 1–15 May 2019 respectively, always by the same observer (R.F.R). This was done to take into account species-specific breeding phenology in the area (early and late breeders)^17^ and minimize bias due to other factors (like weather or disturbance). Sampling was conducted using standardized 10 min point counts^18^ carried out at the central point of the parcel (Fig. 1). As the open terrain allowed for high visibility, a large detection radius was used, and all birds detected within 100 m of the central point were identified and counted. This radius is roughly similar to the one previously used for characterizing bird populations in the region^19^. All counts were carried out in the first four hours after sunrise and in the last two hours before sunset, with none in heavy or persistent rain, or in strong wind conditions. To estimate bird species richness and occurrences in each parcel, we pooled the data from the two counts. Species-level analyses focused on the six most common species, which occurred in > 30% of the parcels (see Supplementary Table S1). In addition to presence/absence, we also estimated population densities, using the count which yielded the highest estimate of density for each species (assuming this is the best indicator of population density, given the potential phenology and detectability biases above mentioned). Bird densities were based on the number of males simultaneously detected and expressed as breeding pairs/10 ha or males/10 ha (in the case of Little Bustard *Tetrax tetrax* and Common Quail *Coturnix coturnix*). Categorization to the genus level was made for the Crested and Thekla larks (*Galerida cristata* and *G. theklae)* due to difficulties in correctly identifying all individuals of these two very similar species in the field.

Vegetation height and cover were measured once in each parcel, between April 22 and May 6. Vegetation height was estimated in a set of ten 3 m radius plots defined inside the 100 m buffer (Fig. 1). In each plot, ten measurements of vegetation height were taken at random locations, for a total of 100 measurements per parcel. Vegetation height was measured using a 50 cm ruler and was defined as the highest point of vegetation projection within 3 cm of the ruler^20^. All values were estimated to the nearest half centimeter. When no vegetation was present (bare soil, soil litter, rocks or animal dung) the height was set to zero (0) but these measurements were not considered to estimate the mean height of the sward. Vegetation cover was measured inside a 50 × 50 cm quadrat placed at each of the ten grid points, by visual estimation to the nearest 5% of the percentage of the quadrat area covered by vegetation^21^ (Fig. 1). Vegetation height and cover measurements were averaged within each parcel.

### Grazing regime

The number and type of livestock in each parcel as well as the extent of the grazing period since the start of the year (2019) were gathered from interviews (Supplementary Information S1) to land managers during 1–15 May 2019. This information was further validated, and corrected in a few cases, through field checks during regular visits (made at two-week intervals) to the parcels (see “Bird and vegetation data” section from Methods). Three grazing regime indicators were estimated for the whole period (January–May 2019): livestock type (either sheep or cattle), animal density, and grazing pressure. The animal density in each parcel was calculated as the average density (animals per hectare) of any species (regardless of being sheep or cattle) that grazed the parcel during the 5-months period. Stocking Rate translated animal density into livestock unit (LU) per hectare (LU/ha), between January and May, according to the following criteria: one adult bovine = 1 LU; bovine aged < 6 months = 0.4 LU; one adult sheep = 0.15 LU^22^. Using LUs allows the comparison of densities across livestock types after correcting for their relative feeding requirements^23^. Grazing Pressure was estimated as the Stocking Rate times the number of days a number of Livestock Units (LU) spent in a plot (LU/ha × number of days)^24^. The area used for these estimations corresponded to the available area where animals could freely roam, which in many cases was larger than the sampled parcel area, which was often not delimited by fences. The number of days in the parcel was collected mainly from the interviews. However, in some cases the extent of grazing period was expressed qualitatively and thus had to be inferred, from common expressions according to the following criteria: ‘few’ = 5 days, ‘some’ = 10 days, ‘a fortnight’ = 15 days, ‘many’ = 20 days, ‘almost all month’ = 25 days^25^.

### Data analysis

Five explanatory variables describing grazing regime and sward structure (Table 1) were used as predictors of bird species richness, occurrence and abundance at parcel level. The correlation and multicollinearity between them were tested and all presented values of r < 0.70 and of variance inflation factor (VIF) smaller than 3^26^.

**Table 1:** Explanatory variables used to model the effect of grazing regime on birds, and respective descriptive statistics for the 50 sampled parcels.

Variable (unit)	Description	Mean ± SD	Min, Max
Livestock type	Type of livestock that grazed the parcel, either sheep (23 parcels) or cattle (27 parcels)	–	–
Animal density (animals/ha)	Mean number of animals that grazed the parcels during the five months period, per unit of area (ha)	3.0 ± 4.2	0, 22.4
Grazing pressure (LU/ha* days)	Total grazing pressure for the five months (January–May) considered for the analysis	93.8 ± 90.9	0, 337.1
Vegetation height (cm)	Mean vegetation height in each parcel	30.0 ± 12.1	12.2, 64.6
Vegetation cover (%)	Mean percentage of vegetation cover per parcel	83.8 ± 12.8	28.6, 99.8

*SD* standard deviation, *Min* Minimum, *Max* Maximum.

Univariate differences between sheep and cattle parcels, both for bird response variables (species richness, density and occurrence) and for the explanatory variables, were tested using Generalized Linear Models (GLMs). We used a Gaussian error distribution and an identity link for quantitative variables, and a binomial error distribution and a logit-link function for occurrence data^26^.

A Structural Equation Modelling (SEM) approach was then used to investigate how grazing regime directly and indirectly affects the occurrence and density of birds. SEM are probabilistic models that hypothesize a causal network with multiple variables that can appear as both predictor and response variables^27^, allowing to look at both direct and indirect effects. We performed a confirmatory-exploratory path analysis^28^ in the form of a piecewise SEM conducted in the R software^29^, using the package “piecewiseSEM”^27^. In piecewise SEM the network is translated as a set of linear equations which can be evaluated individually, using R^2^^30^. The goodness-of-fit of the entire model was quantified by a directed separation test (“d-separation test”), which tests the assumption that all variables are conditionally independent, i.e. that there are no missing relationships among unconnected variables^27,30^.

**Figure 2:**
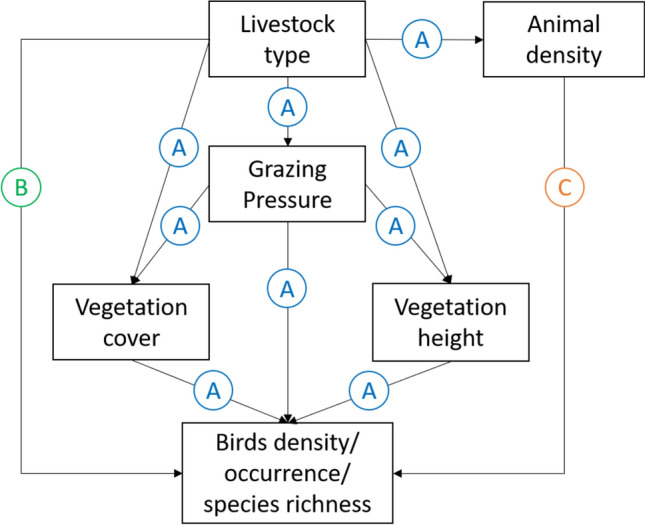
Theoretical model of the confirmatory-exploratory path analysis, where (A) represents the paths of the indirect effect of livestock type via impacts on vegetation structure; (B) represents the livestock type specific direct effects; (C) represents the direct effect of animal density through disturbance impacts on birds. For detailed information of model construction, see Supplementary Information S2.

We started by building a theoretical model of our system (Fig. 2) based on previous literature and knowledge about birds and grasslands (See Supplementary Information S2 for more details on model construction). In short, the model states that the impacts of grazing regime on birds can occur: (A) indirectly, via the impacts of grazing pressure and potentially associated (non-measured) management decisions (e.g. fertilizer use or pasture improvement) on vegetation structure (vegetation height and cover); (B) directly, through the effect livestock-specific (sheep or cattle) behavior (trampling patterns, impacts of feeding mode on food resources for birds, potential egg predation) on birds; or, (C) directly through the disturbance impacts of animal density, expressed as number of herbivores spread over the area, irrespective of livestock type, on birds (Fig. 2). We considered all paths as significant if they had a p-value < 0.1. This threshold was used assuming it could indicate the existence of an effect, even if not significant at the traditional 0.05 level given the relatively low sample sizes. Other authors have used a similar approach in a SEM context (e.g. Sanz-Pérez et al.^11^). Moreover, we used the d-separation test from piecewise SEM output to evaluate our theoretical model and identify eventual significant paths not considered initially.

After obtaining the final model for species richness and for the occurrence and density of each species, we estimated the standardized model parameters (expressed as mean ± standard error; SE) of causal effects. Effect estimates were used to calculate the strengths of direct and indirect effects between variables in the system. Indirect effects were described as a predictor variable (P1) having an effect on the response variable (R) through a simultaneous response and predictor variable (P2), P1 → P2 → R^31^. All statistical analyses were performed within “R” software environment, version 4.0.2^29^.

## Results

### Grazing regime and vegetation structure

**Figure 3:**
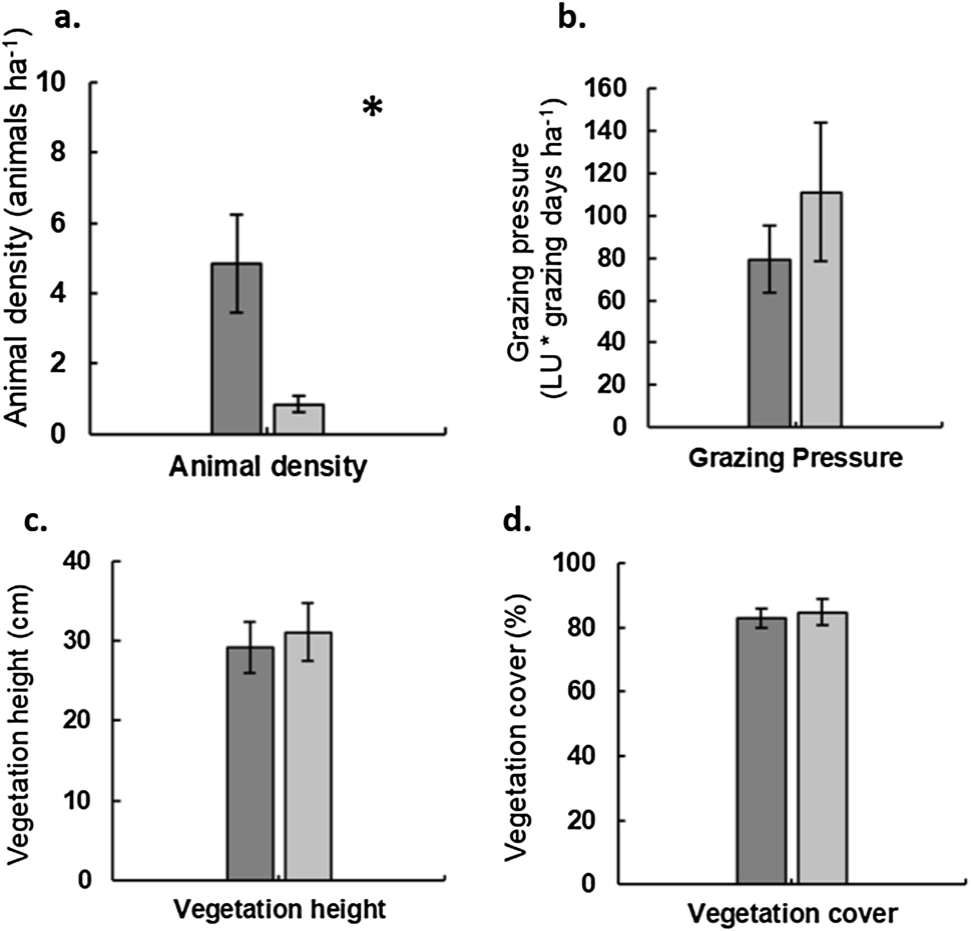
Comparison between sheep (dark grey) and cattle (light grey) grazed parcels for (**a**) Grazing pressure (LU * grazing days/ha); (**b**) Animal density (animals/ha); (**c**) Vegetation height (cm); (**d**) Vegetation cover (%). Values represent average and 95% confidence intervals (shown as vertical black lines). *Highlights significant differences.

Animal density was significantly higher (GLM, *p* < 0.01) in sheep (4.8 ± 1.39 animals/ha) than cattle (0.8 ± 0.24 animals/ha) parcels (Fig. 3a). Grazing pressure was not significantly different between sheep and cattle parcels (GLM, *p* = 0.22) in spite of the trend for higher values in the latter (Fig. 3b). There were also no significant differences between livestock types regarding vegetation height (GLM, *p* = 0.61) and cover (GLM, *p* = 0.62) (Fig. 3c,d).

### Bird species richness and abundance patterns

A total of 25 bird species were observed in the studied parcels, with frequencies of occurrence ranging from a single parcel to all parcels (Supplementary Table S1). Ca. 70% of the species occurred in both livestock systems, and species registered in just one system type had low prevalence (see Supplementary Table S1 for details). The most prevalent species were Corn Bunting *Emberiza calandra* (100% of parcels), *Galerida* spp. (82%), Calandra Lark *Melanocorypha calandra* (82%), Common Quail (58%), Zitting Cisticola *Cisticola juncidis* (50%) and Little Bustard (46%). These six species occurred in both livestock systems and accounted for 86% of all the registers. Another eleven species were present in both cattle and sheep parcels (details in Supplementary Table S1).

**Figure 4:**
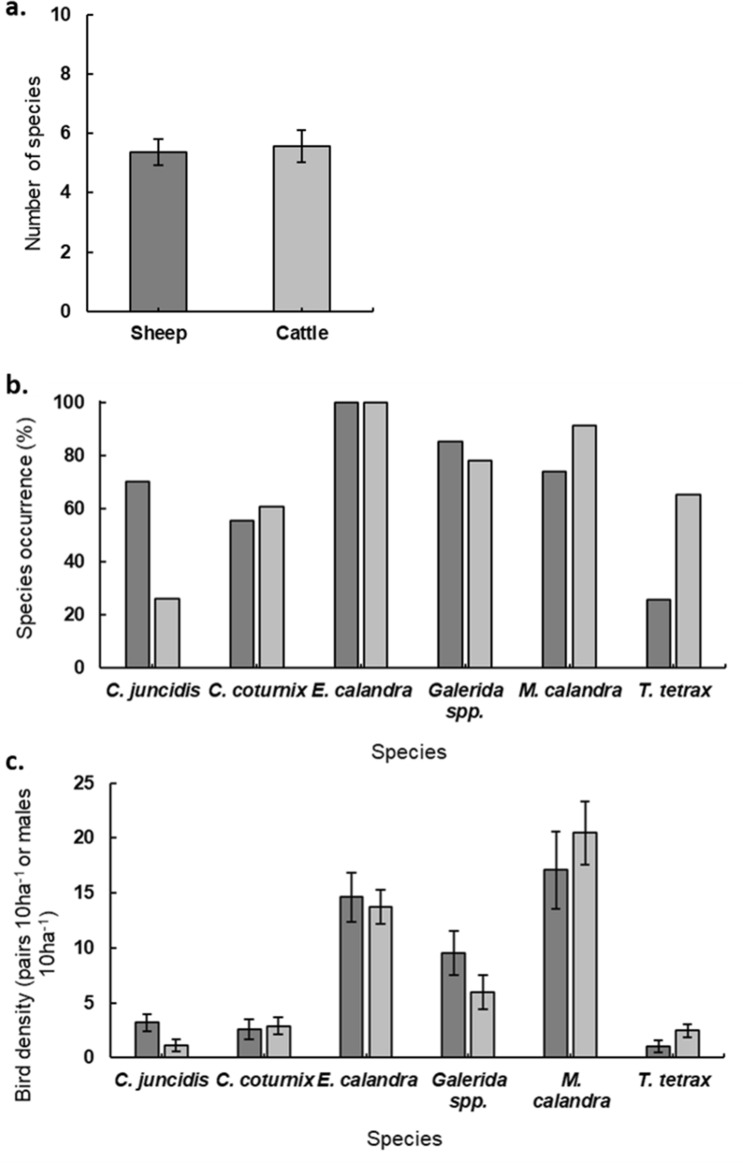
Comparison between sheep (dark grey) and cattle (light grey) grazed parcels for (**a**) Species richness (average number of species in each parcel); (**b**) Occurrence of bird species (% occurrence); (**c**) Average density of bird species (pairs/10 ha or males/10 ha). 95% confidence intervals shown as vertical black lines. *Highlights significant differences.

Species richness was very similar between sheep ($\overline{x }$ = 5.4 ± 0.45) and cattle ($\overline{x }$ = 5.6 ± 0.53) parcels (GLM, *p* = 0.70) (Fig. 4a). There was a higher prevalence and abundance of Zitting Cisticola in sheep pastures (GLM_density_, *p* = 0.01; occurence, *p* < 0.01) and of Little Bustard in cattle parcels (GLM_density_, *p* = 0.03; occurence, *p* < 0.01) (Fig. 4b,c), while no significant univariate effects of livestock type were found for the other species.

### Modelling the effects of grazing regime on birds

SEM results (Table 2) confirmed the higher animal densities in sheep parcels (β = − 0.5, p < 0.01). Also, we found a negative effect of grazing pressure on vegetation height (β = − 0.4, p = 0.01). As for response variables, there were no significant effects on species richness (Supplementary Fig. S2l), but there were several significant effects on individual species occurrences or abundances (Table 3).

**Table 2:** Structural equation model (SEM) parameter estimates (β) and standard errors (SE) for management and vegetation variables (response variables paths and results shown in Supplementary Materials).

Response	Predictor	Unstandardized β		Scale—standardized β	p-value
		Estimate	SE		
Animal density	**← Livestock type**	**− 3.99**	**1.06**	**− 0.48**	**< 0.01**
Grazing pressure	**← **Livestock type	31.6	25.65	0.18	0.22
Vegetation cover	**← **Livestock type	2.44	3.72	0.10	0.52
Vegetation cover	**← **Grazing pressure	**− **0.02	0.02	**− **0.13	0.37
Vegetation height	**← **Livestock type	3.34	3.33	0.14	0.32
Vegetation height	**← Grazing pressure**	**− 0.05**	**0.02**	**− 0.35**	**0.01**
Animal density	**↔ Grazing pressure**	**0.51**	**NA**	**NA**	**< 0.01**
Vegetation height	**↔ Vegetation cover**	**0.38**	**NA**	**NA**	**< 0.01**

Double-headed arrows indicate correlated errors and are shown in the column for unstandardized estimates. Significant effects are highlighted in bold.

**Table 3:** Standardized effects of the significant paths for the density and occurrence of each target species (Supplementary Fig. S2): Zitting Cisticola (CZ), Common Quail (CQ), Corn Bunting (CB), *Galerida* spp., Calandra Lark (CL) and Little Bustard (LB).

Species	Path	Density		Occurrence	
		Effect	R^2^	Effect	R^2^
Zitting Cisticola	Livestock type → ZC	− 0.48***	0.37	− 0.59***	0.43
	Vegetation height → ZC	0.47***		0.45**	
	*Grazing pressure *→* Vegetation height *→* ZC*	− *0.16*		− *0.16*	
Common Quail	Vegetation cover → CQ	n.s	n.s	0.36*	0.13
Corn Bunting	Vegetation height → CB	0.54***	0.33	–	–
	*Grazing pressure *→* Vegetation height *→* CB*	*− 0.19*		–	–
*Galerida* spp.	Livestock density → *Galerida* spp.	0.38**	0.16	n.s	n.s
Calandra Lark	Vegetation height → CL	− 0.31	0.10	n.s	n.s
	*Grazing pressure *→* Vegetation height *→* CL*	*0.11*		n.s	
Little Bustard	Livestock type → LB	0.48***	0.20	0.60***	0.27
	Grazing pressure → LB	−0.34**		n.s	

Standardized path coefficients are shown according to the criteria: ***p < 0.01; **0.01 < *p* < 0.05; *0.05 < *p* < 0.10; n.s. is used for paths with p > 0.1. The indirect effects are shown in italic and were obtained by multiplying the partial standardized path coefficients. Since the Corn Bunting was present in all parcels, the occurrence model was not calculated.

Direct effects of livestock type included the positive association of Zitting Cisticola with sheep (β_density_ = − 0.5, *p* < 0.01; β_ocurrence_ = − 0.6, *p* < 0.01) (Table 3 and Supplementary Fig. S2a,b), and the positive association of Little Bustard with cattle (β_density_ = 0.5, *p* < 0.01; β_ocurrence_ = 0.6, *p* < 0.01) (Table 3 and Supplementary Fig. S2j,k). Animal density showed a positive effect on *Galerida* spp. density (β = 0.4, *p* = 0.05), but no significant effects on other species (Table 3; Supplementary Fig. S2f,g).

The only significant effect of vegetation cover was a positive relation with the prevalence of Common Quail (β_ocurrence_ = 0.4, *p* = 0.08) (Table 3 and Supplementary Fig. S2d). Half the species were influenced by vegetation height (Table 2), with positive effects on Zitting Cisticola (β_density_ = 0.5, *p* < 0.01; β_ocurrence_ = 0.5, *p* = 0.02) and Corn Bunting (β_density_ = 0.5, *p* < 0.01) (Table 3 and Supplementary Fig. S2e), and negative on the density of Calandra Lark (β_density_ = − 0.3, *p* = 0.07) (Table 3 and Supplementary Fig. S2h). The Little Bustard was the only species directly affected (negatively) by increasing grazing pressure (β_density_ = − 0.3, *p* = 0.04) (Table 3 and Supplementary Fig. S2j,k).

## Discussion

Our results showed that the shift from sheep to cattle grazing systems in Mediterranean grasslands did not have major impacts on overall breeding bird species richness. However, there were some species-specific responses influenced by the type of livestock and animal density (both impacting stocking rates and grazing pressure) which were dependent on the livestock system. By analysing the direct and indirect paths through which management decisions are expected to impact on bird populations, we found that grazing pressure was a key driver of the observed responses, which has implications for policy recommendations.

### Changes in grazing regime and vegetation structure

Our results corroborate previous findings that the transition from sheep to cattle grazing systems have clear implications for stocking rates^8,12^. However, an impact on grazing pressure was not observed. Although animal density was higher in sheep than in cattle parcels, grazing pressure tended to be similar, as the cattle feeding requirements are higher than that of sheep due to their larger size and associated nutritional requirements^22^.

Contrary to expectations, no direct effects of livestock type were observed for vegetation height and cover. Sheep and cattle have distinct diets and feeding behaviours^32,33^, with the former usually leading to shorter and uniform swards, and the latter usually promoting structural heterogeneity, with patches of lower and taller vegetation^33,34^. However, the fact that shorter swards were associated to higher grazing pressure, and the latter was tendentially higher in cattle parcels, might have minimized expected differences in vegetation structure.

### Effects of grazing regime on birds

Overall, species richness was similar in sheep and cattle grazed parcels, with all but the least prevalent species occurring in both parcel types. This was expected, as both parcel types shared the same habitat and 68% of the species identified. It also suggests that the studied grazing regime changes are not likely to drastically change farmland bird assemblages at the regional level. Previous studies also did not find differences in bird species richness associated with different livestock management in grasslands^10^.

**Figure 5:**
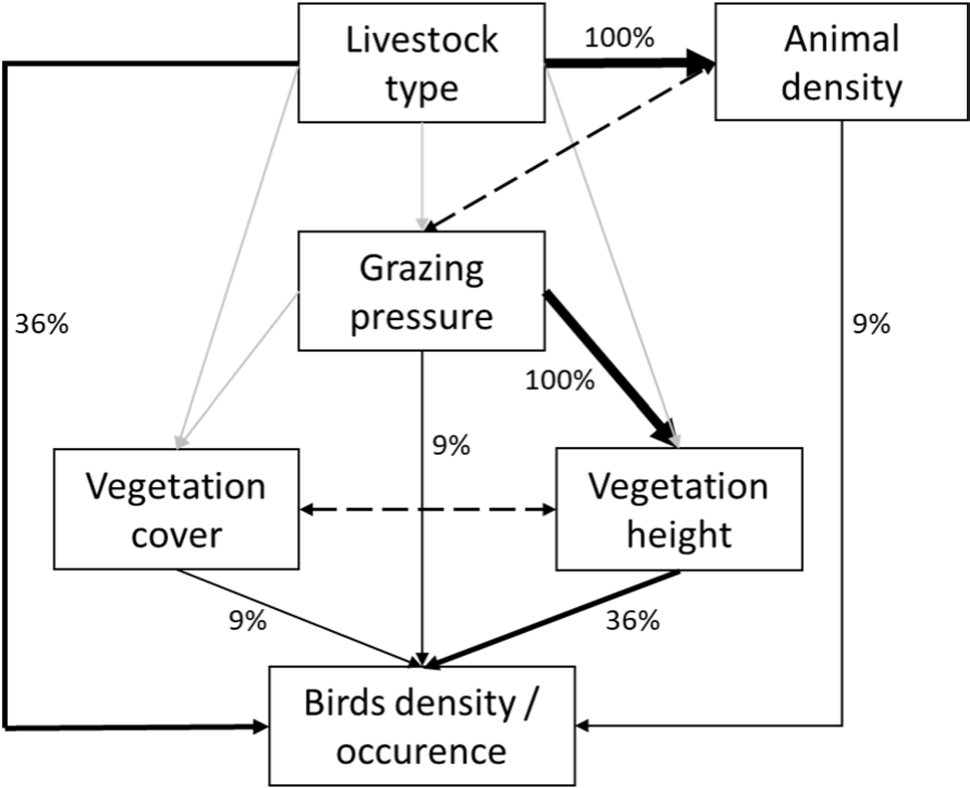
Summary of SEM results for the six bird species density and occurrence, in a total of 11 SEMs. Thickness of the paths is proportional to the number of times that path was significant (< 0.1), regardless of the direction (positive or negative) of the effect.

A direct effect of livestock type on birds was registered in 36% of the models (Fig. 5). Little Bustards’ positive relation with cattle grazed parcels may be related to food resources availability, mainly beetles and other invertebrates, which previous studies reported to be more abundant in cattle grazed fields^35^, and in more heterogeneous fields favouring the occurrence of both males and females^36^. Little Bustard preference for cattle pastures (Fig. 4) was also reported by Reino et al.^13^ for grazed fallow fields in the same region. Zitting Cisticola showed an opposite trend, with a positive response towards sheep grazed parcels. This finding is hard to explain as the species prefers taller swards^20^, which were not directly associated to any specific type of grazer. Other non-measured features might explain this result, such as the likely existence of more cereal fields, a preferred habitat for the species, in the vicinity of sheep parcels (because sheep are associated to the traditional system including cereals), the amount of hedges and tree lines or grazing rotations, previously found to influence the species’ abundance^10^.

Previous studies also reported a preference of Calandra Lark for cattle parcels^10^, a tendency (albeit non-significant) shown in our study. In contrast with our results, previous studies found associations of Corn Bunting to livestock type, although with contrasting patterns, some revealing preference for cattle^13^ and other for sheep^10^.

Positive effects of increasing vegetation height were observed for Zitting Cisticola and Corn Bunting. Both species are usually associated with cereal fields^37^ and tend to select fields with higher vegetation and a low proportion of bare ground^20,38^. This preference can be related to breeding and nesting sites availability. In contrast, Calandra Lark showed a negative relation to vegetation height, as previously documented for this species favouring bare soils^20,39^.

Higher grazing pressure was detrimental to Zitting Cisticola, Corn Bunting and Little Bustard, either direct or indirectly (through vegetation height). Grazing pressure is a key factor influencing species occurrence and breeding success in grassland birds, as it determines not only vegetation structure that provides cover and food availability, but also disturbance levels, which is particularly important during spring because of nest trampling^10,14^. The Little Bustard was directly influenced by grazing pressure, which is in line with previous studies showing its sensitivity to high grazing pressure, possibly because it increases disturbance and exposure to predation during the breeding season^36^. Moreover, male Little Bustards have very specific requirements of vegetation structure during the breeding season, preferring intermediate vegetation height (between 20 and 30 cm), which simultaneously provide concealment against threats and visibility for courtship^40,41^. As such, high grazing pressure likely prevents the development of a suitable sward structure for the species.

Other significant effects included a positive relation between vegetation cover and Common Quail occurrence, which is in line with previous studies suggesting that this species prefers dense and tall swards such as cereal fields^37^. Animal density had a positive effect on *Galerida* spp. density. The fact that we grouped two distinct species of *Galerida* larks (Crested and Thekla larks) can be a confounding factor while interpreting the results, since each species has different habitat requirements^37^. However, the former, usually associated with flat and human-disturbed areas, is much more abundant than the latter, which is more associated with shrublands in hilly areas^42^. This can help explain the positive effect of animal density (likely associated to higher disturbance) on *Galerida* spp.^37^.

## Conclusions

Our study suggests that grazing pressure is the main path through which grazing regime has detrimental effects on several bird species in Mediterranean grasslands. Although we did not find a significant effect of livestock type on grazing pressure in our sampled parcels, other studies provide evidence that the ongoing transition from sheep to cattle systems in the region is reflected in a large increase in stocking rates with likely implications for grazing pressure^8^. This trend is caused by existing CAP subsidies keeping cattle payments partially or fully coupled^7^, meaning that financial support for farmers is proportional to the number of cattle they have. We therefore propose that this policy should be revised, decoupling the subsidies and implementing an area-based payment system limiting the maximum allowed grazing pressure.

Our results also suggest that at the landscape level, maintaining a mix of sheep and cattle grazed fields could be beneficial for maintaining bird diversity, since some species are more associated with a particular grazing regime.

## Supplementary Information

Supplementary Information.

## Data Availability

All supplementary information can be downloaded from the journal’s website.
