# Supplementary material for: Impacts of sheep versus cattle livestock systems on birds of Mediterranean grasslands
Source: Sci Rep. 2021 May 24;11:10827. doi: 10.1038/s41598-021-89975-x (PMC8144398; doi:10.1038/s41598-021-89975-x)
Supplement: Supplementary file 1 — Supplementary Information. [file 41598_2021_89975_MOESM1_ESM.pdf]

Impacts of sheep versus cattle livestock systems on birds of Mediterranean grasslands

Rita F. Ramos, João A. Diogo, Joana Santana, João P. Silva, Luís Reino, Stefan Schindler, Pedro Beja, Ângela Lomba, Francisco Moreira

Supplementary Information S1

Landowners interviews

Along with the bird surveys and vegetation sampling, we interviewed landowners or managers of our target parcels. With these interviews, which occurred in the first fortnight of May 2019, we intended to better understand the history and management of each land plot during the previous 5 years.

We focused on livestock type, number, and extent of grazing period, with an emphasis on the previous agricultural year (from July 2018 to June 2019). Additionally, we also inquired about the livestock patterns for the last 5 years, i.e. if the type of livestock changed or if the number of animals was constant or varied and how much.

Of the selected 50 parcels, we were able to contact and interview 36 land managers, corresponding to 47 parcels. As stated in section 2.3 of Methods, we used the information from the interviews to estimate 3 indicators regarding livestock, namely type, density and grazing pressure. Since we performed field checks to every parcel every two weeks from January 2019 until May 2019, we were able to validate the gathered information. Some of the interviewed reports were flawed or discrepant from what was observed on the field. In such cases, each situation was individually analyzed and corrected using the information from both interviews and site visits.

Supplementary Information S2

Path construction

In order to understand how two different grazing regimes, namely sheep and cattle grazing, may affect grassland birds we used path analysis based on Structural Equation Models (SEM), as it allows us to look for direct and indirect effects of grazing regimes on bird density and presence[1].

We start by choosing our variables of interest (see methods section) and then generated a theoretical model[2] (Figure 2) with all relevant paths being based on our general predictions and previous knowledge. Our model can be summarized in 3 main groups, which account for the direct or indirect effect of livestock on bird species density and occurrence.

The first group accounts for the indirect effect of livestock type on birds, via the impacts of the resulting grazing pressure and other associated management decisions (e.g. fertilizer use or improved pastures) on vegetation structure (vegetation height and cover) (A). The second and third groups account for the direct effect of livestock on birds, either through the effect livestock-specific (sheep or cattle) behavior (trampling patterns, impacts of feeding mode on food resources for birds, potential egg predation) on bird abundance (B) or via disturbance

impacts of livestock on birds (C). Bellow, we describe the supporting theory that allowed us to structure our model.

A) Influence of livestock type indirectly via the impacts of the resulting grazing pressure and associated management on vegetation structure

A link is expected between the type of livestock (sheep or cattle) and vegetation cover and height, either because the resulting grazing pressure might be different, and/or there are differences in associated management (e.g. fertilizer use, improvement of pastures)[3,4] (Supplementary Fig. S1, path A). Moreover, from a management point-of-view and assuming that different types of livestock have different requirements, the animal density on a given parcel might be directly affected by the type of livestock, for example if sheep parcels tend to hold a higher number of animals than a cattle parcel (Supplementary Fig. S1, path A).

Grazing pressure is a measurement of livestock pressure on the pasture based on a widely used measurement of livestock management: the stocking rate[5]. However, this variable also considers the mean number of days, during the studied period, that animals grazed the parcel[6], allowing for a more accurate index of livestock effect on the parcel. The stocking rate and, consequently, the grazing pressure measurements are obtained considering the livestock units (LU) conversation of each livestock type. The LU is a management unit that allows to compare different livestock types and is usually derived in terms of relative feed requirements[7,8]. The conversion ratios are generally based on metabolised energy requirements of an adult cow and in Portugal is converted as follow: adult bovine = 1 LU, young bovine (less than 6 months) = 0.4 LU and adult sheep = 0.15 LU[9]. The stocking rate and the grazing pressure calculations are, consequentially, translated as LU per hectare (LU/ha). Considering these conversions, the livestock type is restricting directly the grazing pressure in each parcel, since 1 adult bovine is equivalent to 6.7 adult sheep. Grazing pressure, which directly depends on the management decisions regarding the LU/ha and the number of grazing days, can likely affect bird species occurrence and densities, since it can significantly alter vegetation and soil features[10].

Regarding vegetation, both livestock type and grazing pressure will influence vegetation structure (Supplementary Fig. S1, path A). Livestock type has a direct effect on vegetation, since the trampling effect and feeding behaviour of cattle and sheep are significantly different[11,12], and those differences can shape vegetation structure and limit vegetation height. Additionally, since sheep and cattle have different feeding requirements, expressed in livestock units, and management decisions like the number of days which a given herd graze a parcel, it is expected that grazing pressure also conditions vegetation[3,4].

Since vegetation structure may influence food availability, nesting sites occurrence and refuge, which are three main ecologic features for grassland birds and passerines[10,13], we expect that vegetation structure have an impact on bird's occurrence and densities, either by vegetation cover or vegetation height. For instance, vegetation cover, plays an important role in providing a heterogeneous landscape, with patches with more vegetation where birds can incubate their eggs and open patches where they can display, forage and look for predators[14,15,16,17]. In addition, moderate to higher vegetation allow for more nesting sites[18,19], while lower vegetation can benefit mating rituals (eg. Little bustards displays[20]).

B) Influence of livestock type directly through other non-measured species-specific (sheep or cattle) effects on birds

Livestock type is likely to influence the occurrence of some bird species due to the different behaviour of cattle and sheep. These differences can significantly alter the habitat, either by promoting different food sources (eg. higher invertebrate abundance in cattle dung)[10,21,22], altering soil composition[4] and causing different intensities of trampling[23] or even nest predation[24].

C) Influence of livestock directly through the disturbance impacts of livestock on birds, regardless of livestock type

The presence of livestock at different densities (regardless of being sheep or cattle) can have a direct impact on bird species densities and occurrence[11,12] (Supplementary Fig. S1, path C). In critical periods, such as breeding season, the impact of livestock disturbance on bird species presence and occurrence can be a serious threat, as a higher animal density can increase the probability of trampling of the nests[11,14,25,26]. However, disturbance can also have a positive impact on more generalist species, which can adapt to highly disturbed habitats[27].

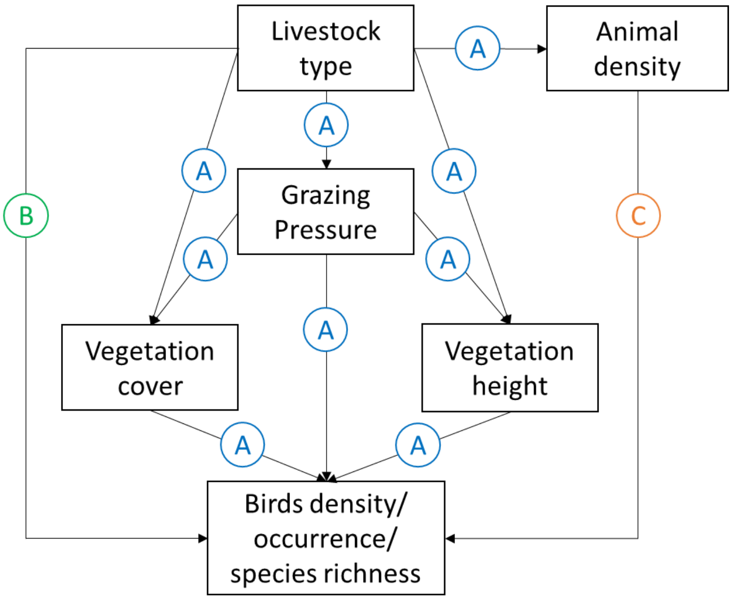

Supplementary Figure S1 – Theoretical model of the confirmatory-exploratory path analysis, where A) represents the paths of the indirect effect of livestock type via impacts on vegetation structure; B) represents the livestock type specific direct effects; C) represents the direct effect of animal density through disturbance impacts on birds.

Supplementary Information S3

Additional results

Supplementary Table S1 – Number of individual detections of bird species during the point counts. Grey background indicates the species with frequency of detection > 30%, for which the density and occurrence analysis were performed.

Species	Sheep parcels		Cattle parcels		Total	
	**Nº individuals**	**Presences**	**Nº individuals**	**Presences**	**Nº individuals**	**Presences**
Emberiza calandra	126	27	100	23	226	50
Melanocorypha calandra	146	20	148	21	294	41
Galerida spp.	81	23	43	18	124	41
Coturnix coturnix	22	15	21	14	43	29
Cisticola juncidis	27	19	8	6	35	25
Tetrax tetrax	11	8	20	15	31	23
Hirundo rustica	22	5	16	6	38	11
Sturnus unicolor	4	3	14	3	18	6
Carduelis carduelis	11	3	3	3	14	6
Upupa epops	3	3	3	3	6	6
Falco naumanni	3	3	3	2	6	5
Alectoris rufa	3	3	2	2	5	5
Calandrella brachydactyla	4	3	1	1	5	4
Corvus corone	1	1	6	2	7	3
Saxicola rubicola	3	2	1	1	4	3
Passer domesticus	2	2	1	1	3	3
Bubulcus ibis	0	0	3	2	3	2
Merops apiaster	0	0	2	2	2	2
Circus pygargus	1	1	1	1	2	2
Coracias garrulus	0	0	2	1	2	1
Cyanopica cyanus	2	1	0	0	2	1
Anthus campestris	0	0	1	1	1	1
Ciconia ciconia	1	1	0	0	1	1
Milvus milvus	1	1	0	0	1	1
Pica pica	1	1	0	0	1	1

Supplementary Information S4

Path analysis results

a.

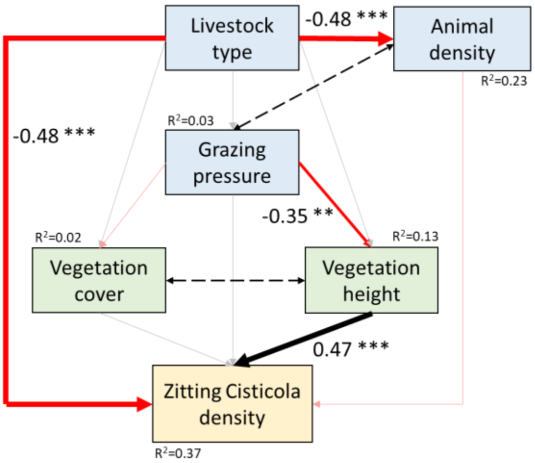

b.

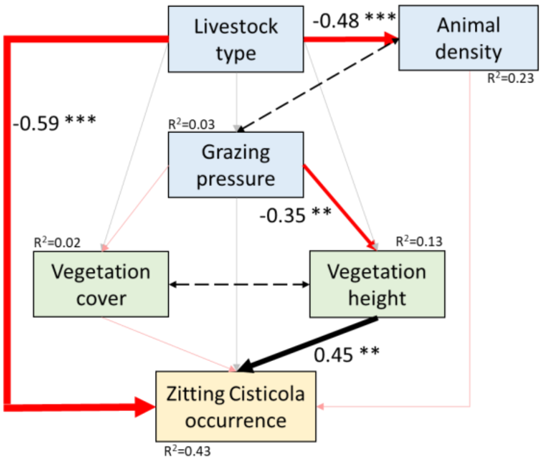

c.

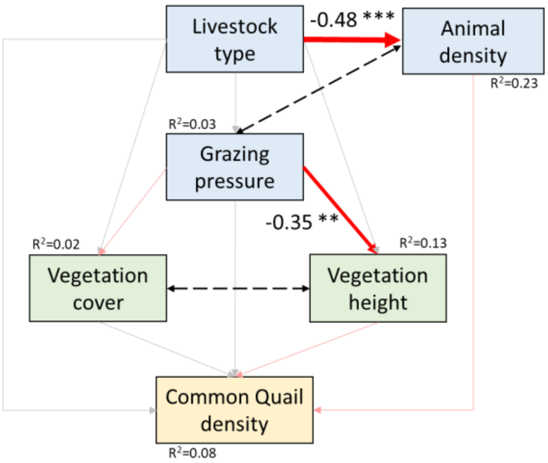

d.

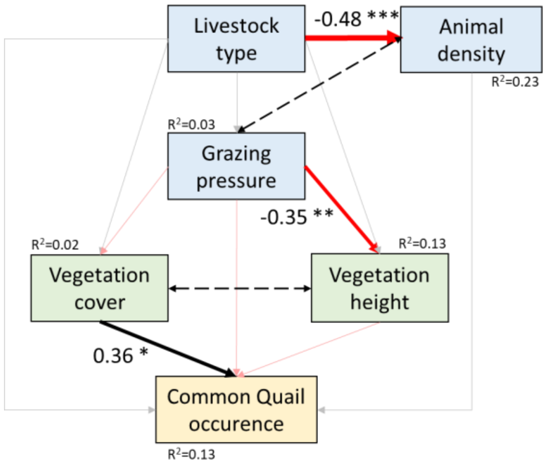

e.

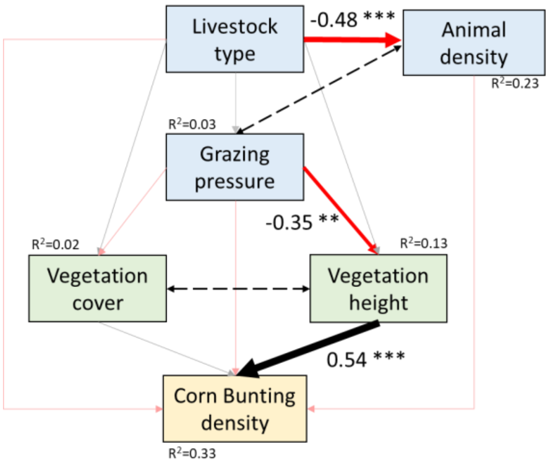

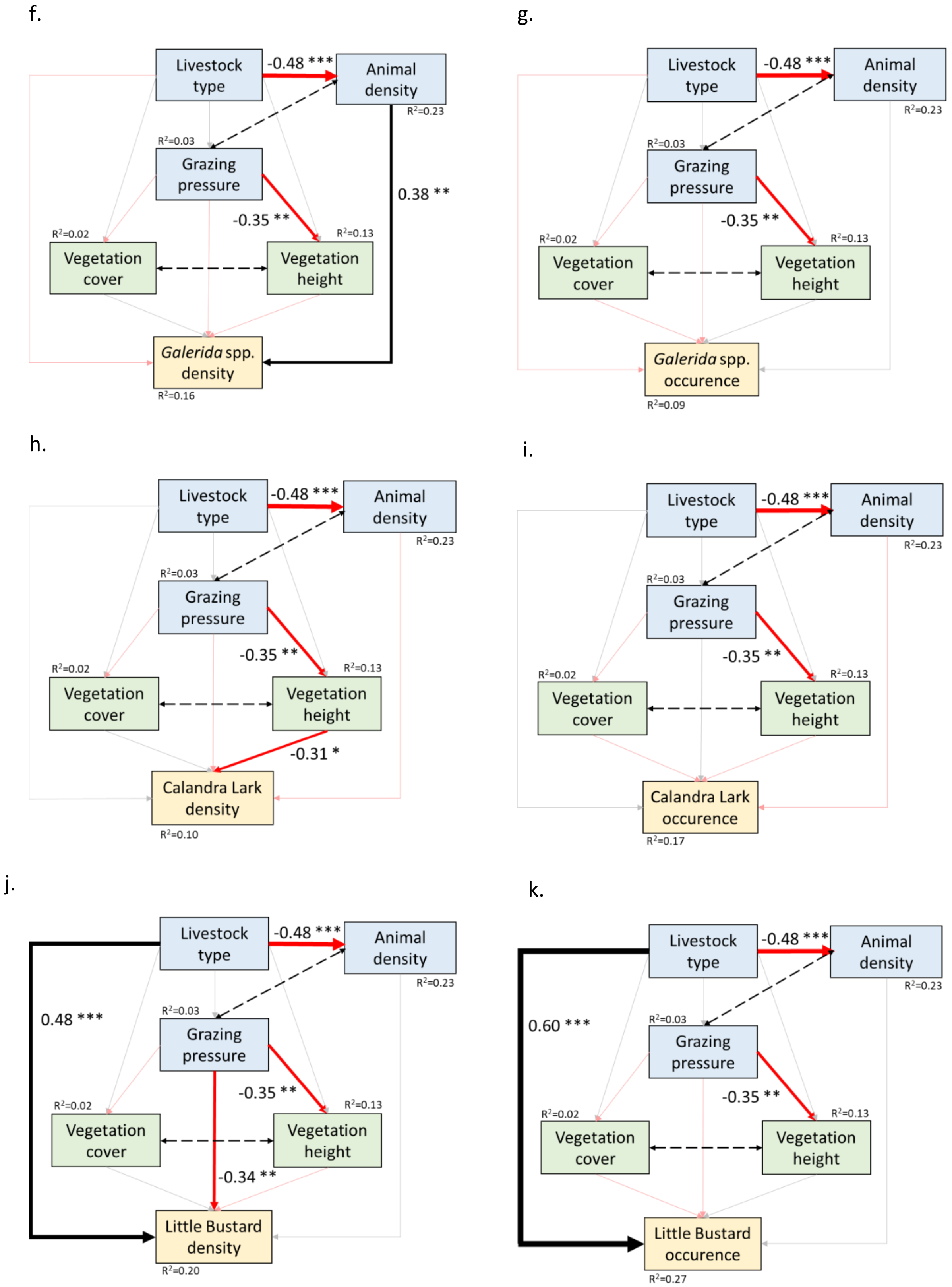

f.
Livestock type
-0.48 ***
Animal density
R²=0.23
R²=0.03
Grazing pressure
0.38 **
-0.35 **
R²=0.02
R²=0.13
Vegetation cover
Vegetation height
Galerida spp. density
R²=0.16
g.
Livestock type
-0.48 ***
Animal density
R²=0.23
R²=0.03
Grazing pressure
-0.35 **
R²=0.02
R²=0.13
Vegetation cover
Vegetation height
Galerida spp. occurence
R²=0.09
h.
Livestock type
-0.48 ***
Animal density
R²=0.23
R²=0.03
Grazing pressure
-0.35 **
R²=0.02
R²=0.13
Vegetation cover
Vegetation height
-0.31 *
Calandra Lark density
R²=0.10
i.
Livestock type
-0.48 ***
Animal density
R²=0.23
R²=0.03
Grazing pressure
-0.35 **
R²=0.02
R²=0.13
Vegetation cover
Vegetation height
Calandra Lark occurence
R²=0.17
j.
Livestock type
-0.48 ***
Animal density
R²=0.23
0.48 ***
R²=0.03
Grazing pressure
-0.35 **
R²=0.02
R²=0.13
Vegetation cover
Vegetation height
-0.34 **
Little Bustard density
R²=0.20
k.
Livestock type
-0.48 ***
Animal density
R²=0.23
0.60 ***
R²=0.03
Grazing pressure
-0.35 **
R²=0.02
R²=0.13
Vegetation cover
Vegetation height
Little Bustard occurence
R²=0.27

l.

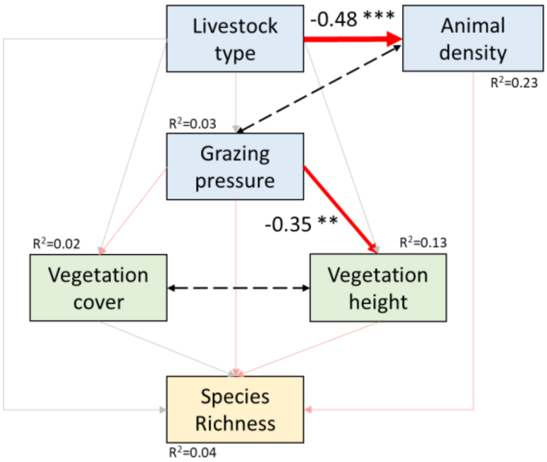

Supplementary Figure S2 - Path diagrams of density and occurrence for Zitting Cisticola (a. and b.), Common Quail (c. and d.), Corn Bunting (e.), *Galerida* spp. (f. and g.), Calandra Lark (h. and i.), Little Bustard (j. and k.) and for species richness (l.).

Conditional R^2 is shown in the top corner of each response variable. Double-headed arrows indicate correlated errors. Thickness of black (positive) and red (negative) paths is proportional to standardized path coefficients. Path transparency is proportional to the *p*-value significance level. Standardized path coefficients are shown according to the criteria: $p < 0.01$***; $0.01 < p < 0.05$**; $0.05 < p < 0.10$*; $p > 0.1$, no value.

Supplementary References

1. Grace, J.B. *Structural Equation Modelling and Natural Systems* (Cambridge University Press, Cambridge, UK/New York, NY, USA, 2006).
2. Grace, J.B. *et al.* Guidelines for a graph-theoretic implementation of structural equation modeling. *Ecosphere*. **3**, 1–44 (2012).
3. Jones, A. Effects of cattle grazing on North American arid ecosystems: a quantitative review. *West. N. Am. Nat.* **60**, 155–164 (2000).
4. Eldridge, D.J., Delgado-Baquerizo, M., Travers, S.K., Val, J. & Oliver, I. Do grazing intensity and herbivore type affect soil health? Insights from a semi-arid productivity gradient. *J. Appl. Ecol*. **54**, 976–985 (2017).
5. Faria, N. Predicting agronomical and ecological effects of shifting from sheep to cattle grazing in highly dynamic Mediterranean dry grasslands. Land. *Degrad. Dev*. **30**, 300–314 (2019).
6. Gonçalves, P., Alcobia, S., Simões, L. & Santos-Reis, M. Effects of management options on mammal richness in a Mediterranean agro-silvo-pastoral system. *Agrofor. Syst*. **85**, 383–395 (2012).
7. Attwood, E.A. & Heavey, J.F. Determination of grazing livestock units. *Irish J. Agric. Res.* **3,** 249-251 (1964).
8. FAO. *Guidelines for the preparation of livestock sector reviews* (Animal Production and Health Guidelines. No. 5. Rome, 2011).
9. DRE. Anexo 2 - Tabela de conversão em cabeças normais (CN). Diário da República, 1.ª série, 41: Portaria n.º 57 http://data.dre.pt/eli/port/57/(2015)/02/27/p/dre/pt/html (2015).
10. Vickery, J.A. *et al.* The management of lowland neutral grasslands in Britain: effects of agricultural practices on birds and their food resources. *J. Appl. Ecol*. **38**, 647–664 (2001).
11. Ausden, M. & Treweek, J. *Grasslands*. In: *Managing Habitats for Conservation.* 197–229 (Cambridge University Press, Cambridge, 1995).
12. Cassidy, L.R. & Kleppel, G. The effect of grazing regime on grassland bird abundance in New York State. *Northeastern Naturalist*. **24**, 86–99 (2017).

13. Fisher, R. & Davis, S.K. From Wiens to Robel: A review of grassland-bird habitat selection. *J. Wildl. Manag.* **74**, 265–273 (2010).
14. Moreira, F. Relationships between vegetation structure and breeding bird densities in fallow cereal steppes of Castro Verde, Portugal. *Bird Study*. **46**, 309–318 (1999).
15. Silva, J.P., Faria, N. & Catry, T. Summer habitat selection and abundance of the threatened little bustard in Iberian agricultural landscapes. *Biol. Conserv*. **139**, 186–194 (2007).
16. Jaster, L.A., Jensen, W.E. & Lanyon, W.E. *Eastern meadowlark (Sturnella magna*). No. 160, In: *The Birds of North America Online* (Cornell Lab of Ornithology, Ithaca, NY, 2012).
17. Buckingham, D. & Peach, W. The influence of livestock management on habitat quality for farmland birds. *Anim. Sci.* **81**, 199–203 (2005).
18. Moreira, F. *et al.* Population trends in the steppe birds of Castro Verde in the period 2006-2011: Consequences of a drought event and land use changes? *Airo*, **22**, 79–89 (2012).
19. Faria, N., Rabaça, J.E. & Morales, M.B. The importance of grazing regime in the provision of breeding habitat for grassland birds: the case of the endangered little bustard (*Tetrax tetrax*). *J. Nat. Conserv*. **20**, 211–218 (2012).
20. Silva, J.P., Palmeirim, J.M. & Moreira, F. Higher breeding densities of the threatened little bustard *Tetrax tetrax* occur in larger grassland fields: Implications for conservation. *Biol. Conserv.* **143**, 2553–2558 (2010).
21. Buckingham, D.L., Peach, W.J. & Fox, D.S. Effects of agricultural management on the use of lowland grassland by foraging birds*. Agric. Ecosyst. Environ*. **112**, 21–40 (2006).
22. Reino, L. *et al*. Effects of changed grazing regimes and habitat fragmentation on Mediterranean grassland birds. *Agric. Ecosyst. Environ*. **138**, 27–34 (2010).
23. Yong-Zhong, S., Yu-Lin, L., Jian-Yuan, C. & Wen-Zhi, Z. Influences of continuous grazing and livestock exclusion on soil properties in a degraded sandy grassland, Inner Mongolia, northern China. *Catena.* **59**, 267–278 (2005).
24. Beja, P. *et al*. Predators and livestock reduce bird nest survival in intensive Mediterranean farmland. *Eur. J. Wildl. Res.*, **60**, 249-258 (2014).
25. Saab, V.A., Bock, C.E., Rich, T.D. & Dobkin, D.S. *Livestock grazing effects in western North America*, In: *Ecology and management of neotropical migratory birds*. 311–353 (Oxford University Press, New York, New York, USA, 1995)
26. Green, R.E. *The management of lowland wet grassland for birds.* 48 pp. (Nature Conservation Council, Peterborough, 1986).
27. Leitão, P.J., Moreira, F. & Osborne, P.E. Breeding habitat selection of steppe birds in Castro Verde: A remote sensing and advanced statistics approach. *Ardeola*. **57**, 93–116 (2010).
